# CD14 overexpression upregulates TNF-α-mediated inflammatory responses and suppresses the malignancy of gastric carcinoma cells

**DOI:** 10.1007/s11010-013-1559-0

**Published:** 2013-01-22

**Authors:** Kang Li, Zeng Dan, Xuejun Hu, Meiduo Ouzhu, Yangjin Ciren, Zhonghua Wang, Jia Wang, Xixia Yang, Yongge Ze

**Affiliations:** 1 Department of Gastroenterology, People’s Hospital of Tibet Autonomous Region, 18# North Lin Kuo Road, Lhasa, Tibet, 850000 China; 2 Physical Examination Center, People’s Hospital of Tibet Autonomous Region, Lhasa, 850000 China; 3 Department of Oncology, People’s Hospital of Tibet Autonomous Region, Lhasa, 850000 China

**Keywords:** CD14, Gastric carcinoma, SGC-7901, NF-κB

## Abstract

CD14 mediates the inflammatory response via recognition of lipopolysaccharide, which has been implicated in *Helicobacter pylori* (*H. pylori*) infections. Increasing evidence has suggested that CD14 status significantly influences the clinical outcome of *H. pylori* infection, which can result in gastric carcinoma. However, there is little evidence regarding the cellular impact and associated molecular basis of CD14 on gastric carcinoma cells. To address this question, we generated a CD14-overexpressing SGC-7901 gastric carcinoma cell line and analyzed the impact of CD14 expression. Our results revealed that cells overexpressing CD14 exhibited antitumor potential, including significantly decreased clonogenic ability, proliferation, metastatic invasion, as well as enhanced apoptosis, suggesting a tumor-suppressive role of CD14 in the cells. Intriguingly, we further discovered that CD14 overexpression activated NF-κB via upregulating its expression and simultaneously stimulating DNA binding activity. Upregulated NF-κB transcriptionally elevated a series of pro-inflammatory cytokines, including TNF-α, IL-1β, IL-6, and IL-12. Together, the current study utilized a CD14-overexpressing gastric cell model to determine the impacts of CD14 upregulation on cell viability, apoptosis, and migration and NF-κB-mediated inflammation.

## Introduction

Gastric carcinoma is the second most common cause of cancer deaths worldwide, accounting for ~738,000 deaths in 2008. Approximately 21,000 new cases of gastric cancer are diagnosed in the United States each year and more than 10,000 deaths are caused by the disease [1, 2]. It is one of the most common malignant tumors in China as well, and was the third leading cause of cancer-related deaths in China in 2005. The incidence of gastric cancer is considered a consequence of the long-term effects of multiple factors and varies significantly by region. Studies have shown that *Helicobacter pylori* (*H. pylori*) infection, diet, smoking, and genetic susceptibility are important factors involved in gastric malignancies [3].

*Helicobacter pylori*, a gram-negative bacterium that infects the human stomach and causes chronic mucosal inflammation, is the primary identified cause of gastric cancer [4]. Epidemiological studies have revealed that *H. pylori* infection is the strongest known risk factor for gastric malignancies and the attributable risk for gastric cancer conferred by *H. pylori* is ~75 % [5, 6]. The pathogenesis of gastric carcinoma is believed to initiate with *H. pylori*-induced chronic superficial gastritis, followed by atrophic gastritis, intestinal metaplasia, dysplasia, which eventually progresses into gastric carcinoma. Although *H. pylori* proteins and the induced epithelial responses clearly influence disease risk, they are not absolute determinants of carcinogenesis. Indeed, the driving force of gastric carcinogenesis appears to be the persistent gastric inflammation. Inflammation intensity and localization determines the risk of gastric carcinoma [7].

The innate immune system modulates the chronic inflammation caused by persistent *H. pylori* infection [8]. Toll-like receptors (TLR) are members of the pattern-recognition receptor family and TLR signaling activates the innate immune system as well as instructs antigen-specific adaptive immunity. It has been shown that variations in innate immunity may influence immune responses and thus contribute to infectious disease diversity [9, 10]. CD14 is a pattern-recognition receptor that plays a key role in innate immunity and directs adaptive immune responses. CD14 is a co-receptor of TLRs that acts primarily by transferring lipopolysaccharide (LPS) and other bacterial ligands from circulating LPS-binding proteins to the TLR4/MD-2 signaling complex. These signals in turn activate transcription factors, mainly nuclear factor-κB (NF-κB), and cytokines [11–14]. Notably, compelling evidence indicates that CD14 levels are closely associated with H. *pylori* disease outcome, suggesting that CD14 may be an important factor for determining the progression of *H. pylori* infection-associated gastric malignancy.

Despite the epidemiological evidence, data regarding the impact of CD14 on gastric carcinoma cells has been rare. Here, we investigate the effects of CD14 on gastric cancer cells using a gastric cancer cell line ectopically expressing CD14.

## Materials and methods

### Cell culture and treatment

The human gastric carcinoma cell line SGC-7901 was obtained from American Type Culture Collection (ATCC). SGC-7901 cells were transfected with pcDNA 3.1-EGFP (empty vector) or pcDNA 3.1-CD14 and subjected to G418 selection. The transfection efficacy was determined by GFP microscopy and CD-14 expression was verified by western blot analyses. Cells were grown in Dulbecco’s Modified Eagle Medium (DMEM; GIBCO, Grand Island, NY) supplemented 10 % fetal bovine serum (FBS; Thermo, Logan, UT) at 37 °C in a humidified atmosphere with 5 % CO_2_ in air. Cells were stimulated with muramyl dipeptide (MDP; Sigma, St. Louis, MO) before further analyses.

### Colony formation assays

Colony formation assays were used to evaluate the impact of CD-14 on the clonogenic ability of SGC-7901 cells. Briefly, cells were seeded in 6-well plates at a density of 1,000 cells/well and cultured for 7 days. Culture media was refreshed every 2–3 days. Colonies containing ≥50 cells were considered representative of clonogenic cells. The clonogenic fraction was calculated by the formula: (number of colonies formed/number of cells seeded) × 100 %. The values presented are the mean from three independent experiments.

### Cell viability assays

Cell viability was measured by a CCK-8 assay. Cells were seeded in 96-well plates at a density of 5 × 10^3^/well, grown for 24 h to allow attachment, and the culture media was replaced with fresh media. Cells were grown for 4 days and cell viability was assessed each day using a CCK-8 kit purchased from Beyotime Institute of Biotechnology (Haimen, China). Plates were analyzed at 450 nm using a 96-well microplate reader. The growth curve was plotted according to the average measurements of five replicates.

### Apoptosis assays

Apoptosis was detected using an Annexin V/Propidium Iodide (PI) dual staining kit purchased from KeyGen Biotech (Nanjing, China). Cells were co-stained with Annexin V and PI and subjected to flow cytometry analyses following the manufacturer’s instructions.

### Western blot analyses

Cells were lysed in RIPA lysis buffer purchased from Beyotime Institute of Biotechnology (Haimen, China) and equal amounts of total protein were separated by 10 % SDS-PAGE. Proteins were electrotransferred to PVDF membranes and incubated with the primary antibodies overnight at 4 °C, followed by incubation with horseradish peroxidase-conjugated secondary antibodies. The sources and dilutions of antibodies are included in Table 1. β-actin was used as an internal control. Immunoreactive bands were visualized using the ECL Western Blotting Detection System (Pierce Inc, Rockford, IL) according to the manufacturer’s instructions.

**Table 1:** Sources and dilutions of primary antibodies

Antibody	Source	Dilution	Incubation condition
NF-kB	Santa Cruz	1:400	4 °C over night
hBD-2	Santa Cruz	1:500	4 °C over night
TNF-α	Bioss	1:500	4 °C over night
IL-1β	Bioss	1:800	4 °C over night
IL-6	Bioss	1:800	4 °C over night
IL-12	Bioss	1:500	4 °C over night
CD14	Santa Cruz	1:300	4 °C over night

*Santa Cruz* Santa Cruz Biotechnology, Inc

### Transwell invasion assays

The impact of CD-14 on cell invasion was measured in Transwell chambers containing 6.4-mm diameter polyethylene terephthalate filters with 8.0-μm pore size (BD Falcon, Bedford, MA). Cells were seeded on filters coated with Matrigel. Culture media supplemented with 1 % FBS was added to both chambers to measure invasion due to CD-14 overexpression. Cells were incubated at 37 °C for 24 or 48 h to allow sufficient time for migration. Cells on the upper side of the filter were mechanically removed and cells that migrated to the lower side of the filter were fixed with formaldehyde, stained with hematoxylin, and photographed. For semi-quantification of cell invasion, 5 fields per well were randomly selected and cells were counted. Images are representative of at least three independent experiments.

### Gel electrophoresis mobility shift (EMSA) assays

EMSA assays were performed to determine the DNA binding activity of NF-κB using a commercial kit (Chemiluminescent EMSA Kit; Beyotime Institute of Biotechnology, Haimen, China) according to the manufacturer’ instructions. Nuclear extracts were first prepared from SGC-7901 cells overexpressing CD-14 and control cells using the extract buffers A and B supplied in the kit following the instructions. Nuclear extracts were incubated with a radioactive oligonucleotide probe that contains the specific recognition sequence for NF-κB (5′-AGT TGA GGG GAC TTT CCC AGG C-3′ and 3′-TCA ACT CCC CTG AAA GGG TCC G-5′). The binding reaction occurred in nuclease-free EMSA/gel-shift binding buffer containing 1 μl of probe and 8 μg of nuclear proteins. The p65-labeled NF-κB antibody (0.2 μg) was added to the super-shifted sample as the positive control. After binding, the samples were separated on a 4 % native PAGE gel and the proteins were transferred and UV-crosslinked for 45 s. Bands were visualized using a probe biotin-labeling kit (BeyoECL Plus, Beyotime Institute of Biotechnology).

### RT-PCR analyses

RT-PCR analyses were performed using the total RNA extraction kit, together with the Script cDNA synthesis kit and 2× Taq PCR Master Mix, purchased from Tiangen Biotech (Beijing, China). Briefly, total RNA was isolated using the Trizol reagent (Gibco) according to the manufacturer’s instructions. Total RNA (1 μg) from each sample was reverse transcribed using oligo dT primers, deoxynucleotide triphosphates (dNTPs), and moloney murine leukemia virus (M-MLV) reverse transcriptase in a total reaction volume of 20 μl. PCR was performed on cDNA using Taq DNA polymerase and dNTPs with the following cycling parameters: 95 °C for 5 min, 95 °C for 20 s, 60 °C for 20 s, and 72 °C for 30 s for 30 cycles and a final extension of 72 °C for 5 min. An aliquot of each reaction mixture was analyzed by electrophoresis on a 1 % agarose gel. Images were obtained after staining with ethidium bromide. The primer sequences used for PCR are included in Table 2. β-actin was used as an internal control.

**Table 2:** Oligonucleotide primer sequences for PCR

Gene	Sequence (5′–3′)	Size (bp)
hBD-2 F	CCT CTTCATA TTC CTG ATG CCT CT	127
hBD-2 R	GGT GCC AAT TTG TTT ATA CCT TCT AG	
TNF-α F	GTC TCC TAC CAG ACC AAG GTC AAC	221
TNF-α R	CAC AGG GCA ATG ATC CCA AAG TAG	
IL-1β F	CCT GGA CTT TCC TGT TGT CTA CAC C	178
IL-1β R	TCT GTC AGG CGG GCT TTA AGT GAG	
IL-6 F	TCA CCT CTT CAG AAC GAA TTG ACA	115
IL-6 R	AGT GCC TCT TTG CTG CTT TCA CAC	
IL-12 F	AGA TGG TAT CAC CTG GAC CTT GGA C	133
IL-12 R	ATG GCT TAG AAC CTC GCC TCC TTT G	
β-actin F	ACG TTG ACA TCC GTA AAG AC	200
β-actin R	GAA GGT GGA CAG TGA GGC	

*F* forward, *R* reverse

### Statistical analyses

Data are presented as the mean ± SD. A student’s *t* test was used to assess statistic significance and *p* < 0.05 was considered statistically significant. All the results were analyzed by means of the SPSS statistical software package (version 12.0).

## Results

CD-14 overexpression causes decreased clonogenic ability and cell viability in SGC-7901 gastric carcinoma cells.

**Fig. 1:**
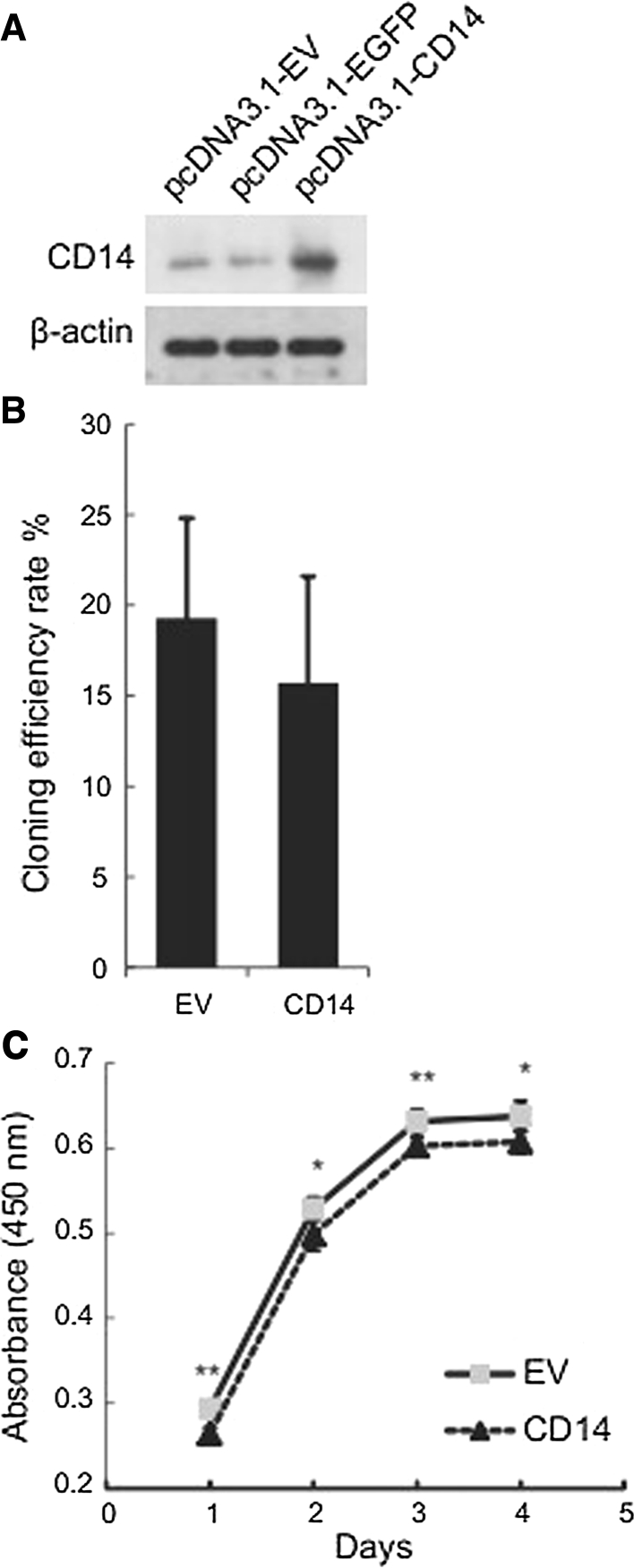
Overexpression of CD-14 minimally affects the clonogenic ability of SGC-7901 gastric carcinoma cells, but decreases cell viability. **a** Western blotting analyses of CD14 ectopic expression in SGC-7901 cells. Cells transfected with EGFP were used as a control. **b** Fraction of clonogenic cells transfected with empty vector (EV) or CD14 overexpression. **c** Cell viability determined by CCK-8 assay. Data are presented as the mean ± SD. *p < 0.05, **p < 0.01 versus control cells

To determine the impact of CD-14 on the cellular behavior of gastric carcinoma cells, we established a SGC-7901 gastric cancer cell line stably overexpressing CD-14. Cells expressing GFP were used as a control (Fig. 1a). We first examined the clonogenic ability of the cells following MDP stimulation by a colony formation assay. We observed a decreased clonogenic fraction in CD-14 overexpressing cells, suggesting that CD-14 suppresses the clonogenic ability of SGC-7901 cells (Fig. 1b). We also assessed cell viability by CCK-8 assay, a colorimetric assay that reflects the mitochondrial activity of the cells. We found that cells overexpressing CD-14 exhibited significantly (*p* < 0.05) reduced cell viability (Fig. 1c), suggesting that the CD-14-mediated inflammatory response diminishes the gastric cancer cell viability.

### CD-14 overexpression increases apoptosis in SGC-7901 gastric carcinoma cells

**Fig. 2:**
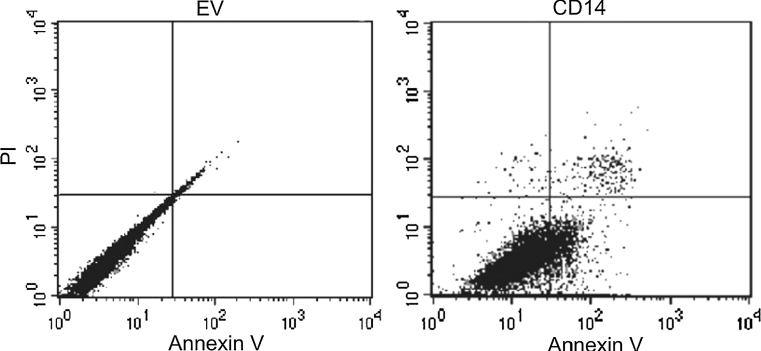
Overexpression of CD14 induces apoptosis in SGC-7901 cells. Cells stably overexpressing CD14 or empty vector (EV) were stimulated by muramyl dipeptide (MDP) before dual staining with Annexin V/PI. Images shown are representative of at least three independent experiments

We next assessed MDP-induced apoptosis in CD-14 overexpressing cells. Apoptotic cells were identified by Annexin V staining and detected by flow cytometry. Upon MDP stimulation, an increase in apoptotic cells was observed in CD-14 overexpressing cells compared with cells expressing empty vector (Fig. 2). These data suggest that the CD-14-mediated inflammatory response tends to induce apoptosis in gastric cancer cells.

### Overexpression of CD-14 suppresses the invasive potential of SGC-7901 gastric carcinoma cells

**Fig. 3:**
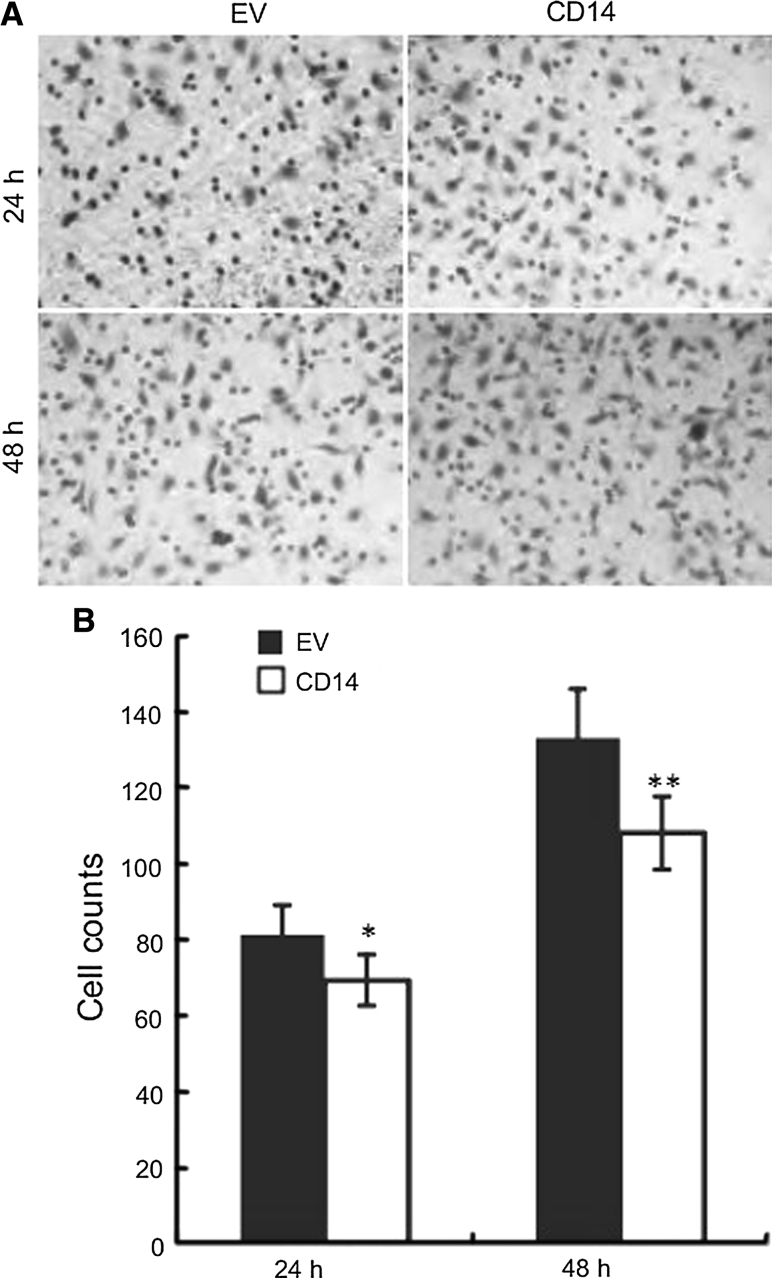
CD14 overexpression decreases the invasive potential of SGC-7901 cells. Cells stably overexpressing CD14 or empty vector (EV) were seeded in the upper wells of pre-coated matrigel transwell chambers. Invasiveness was determined by staining cells that migrated to the lower wells of the chamber. **a** Images shown are representative of at least three independent experiments. **b** Semi-quantification of images shown in (**a**). Five fields per well were randomly selected for cell counts. Data are presented as the mean ± SD. *p < 0.05, **p < 0.01 versus control cells

The invasive potential of cancer cells is critical for cancer metastasis. Therefore, we evaluated the impact of CD-14 on gastric cancer cell invasiveness by transwell invasion assays. Cells were allowed to migrate through matrigel-coated transwell inserts into lower wells containing 10 % FBS for 24 or 48 h. By comparing the number of cells migrating through the matrigel layer, we determined that CD-14 upregulation suppresses the invasive potential of SGC-7901 cells (Fig. 3a, b).

### CD-14 stimulates NF-κB DNA binding activity in SGC-7901 gastric carcinoma cells

**Fig. 4:**
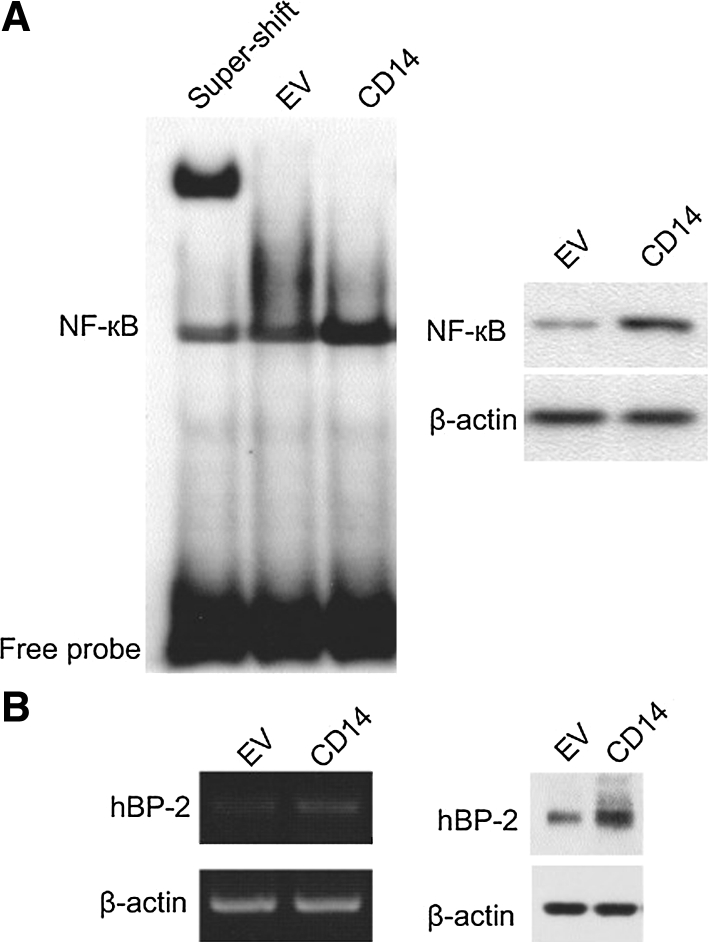
CD14 overexpression stimulates NF-κB activity. NF-κB activity and expression were evaluated in cells stably overexpressing CD14 or empty vector (EV). **a** DNA binding activity of NF-κB measured by EMSA assay. **b** hBD-2 mRNA and protein expression in cells stably overexpressing CD14 (CD14 exp) and SGC-7901 cells (WT)

NF-κB is a major effector of the CD-14-mediated inflammatory response upon *H. pylori* infection. Thus, we examined whether increased expression of CD-14 could activate NF-κB in gastric cancer cells. We first evaluated NF-κB protein expression in cells ectopically expressing CD-14. Compared with control cells, NF-κB protein expression in CD-14 overexpressing cells was approximately twofold higher (right panel, Fig. 4a). In addition, we assessed the DNA binding activity of NF-κB by EMSA assays. We found that in CD-14 overexpressing cells, NF-κB showed a higher fraction of DNA binding protein. Super-shift experiments performed by adding a specific NF-κB antibody further shifted the DNA-binding band, indicating the DNA bound bands observed in our assay were indeed NF-κB (left panel, Fig. 4a). Together, these results suggest increased NF-κB activity. Further, we validated the function of activated NF-κB by determining the mRNA and protein levels of one of its downstream proteins, hBD-2. At both levels, cells overexpressing CD14 showed higher levels of hBD-2 (Fig. 4b), reflecting upregulated NF-κB activity.

### CD-14 overexpression upregulates pro-inflammatory cytokines in SGC-7901 gastric carcinoma cells

**Fig. 5:**
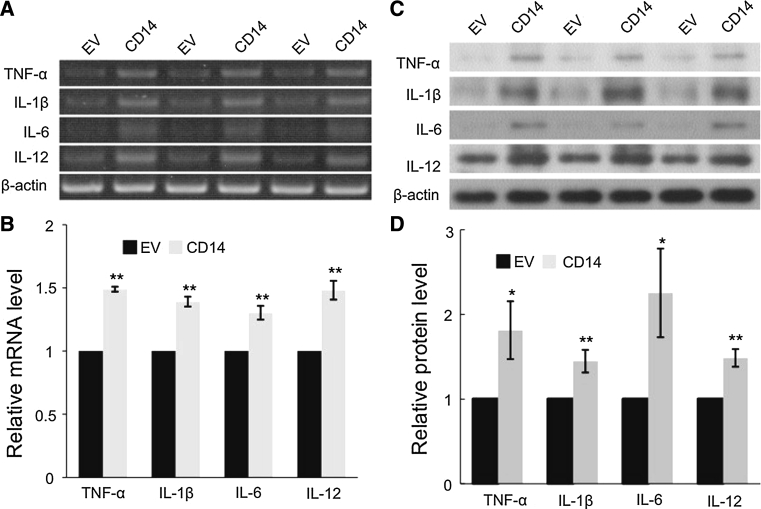
CD14 induces pro-inflammatory cytokine expression in SGC-7901 cells. **a** Representative images of mRNA levels from RT-PCR assay. **b** Semi-quantitation of (**a**) by measuring the band intensities using a densitometer. Data are presented as the mean ± SD. **p < 0.01 versus control cells (EV). **c** Representative images of protein expression from western blot analyses. **d** Semi-quantitation of (**a**) by measuring the band intensities using a densitometer. Data are presented as the mean ± SD. *p < 0.05, **p < 0.01 versus control cells

Activation of NF-κB likely leads to transcriptional upregulation of target genes, including pro-inflammatory cytokines. We measured the mRNA and protein levels of several pro-inflammatory cytokines including TNF-α, IL-1β, IL-6, and IL-12. At both the mRNA (Fig. 5a, b) and protein levels (Fig. 5c, d), these cytokines significantly increased upon MDP stimulation in CD14 overexpressing cells.

## Discussion

*Helicobacter pylori* has been associated with the etiology of gastric cancer. However, the clinical outcome of *H. pylori* infection is highly variable and may be influenced by both microbial and host factors [2, 15]. Thus, only a small fraction of infected individuals develop cancer. Immunogenetic factors play important roles in the progression and severity of inflammation, thereby influencing the clinical outcome of *H. pylori* infection [16]. The evolutionarily conserved innate immunity system is the first-line defense against microbial invasion [17], and this system may modulate chronic inflammation resulting from persistent *H. pylori* infection [8]. The germ line-encoded pattern-recognition receptors are important components of innate immunity and play a central role in pathogen recognition and immune responses. TLR signaling activates innate immunity as well as instructs antigen-specific adaptive immunity [18]. It has been shown that variations in innate immunity may influence immune responses and thus contribute to the diversity of infectious disease [9, 10]. It is well recognized that CD14 is crucial for innate immune responses to bacterial infection as well as in the initiation of cytokine cascades. In addition to being a component of the TLR4/MD-2 complex, CD14 is also involved in TLR2 and TLR6 receptor-mediated signaling [19]. These signaling pathways activate various transcription factors such as IFN, activating protein-1 (AP-1), and NF-κB upon bacterial infection, resulting in the production of pro-inflammatory cytokines and chemokines [20]. Consistently, overexpression of CD14 and TLRs in *H. pylori* infected gastric mucosa has been observed, particularly in gastric tumor tissues [21, 22]. In particular, recent studies have observed that functional CD14 polymorphisms, especially in the promoter motifs, are associated with a higher risk of *H. pylori*-related gastric carcinoma [23, 24]. However, it is important to recognize that the association between CD14 and gastric cancer risk may vary between ethnicities [25]. Therefore, it is crucial to gain more insight regarding the role of CD14 in gastric cancer progression.

In this study, we investigated the cellular impacts of CD14 on gastric carcinoma cells after MDP stimulation. We discovered that CD14 affects several key events including cell viability, apoptosis, migration as well as NF-κB-mediated inflammation response. The mechanisms by which CD14-mediated chronic inflammation leads to epithelial and pre-cancerous lesions remain unclear. Previous studies have suggested it may include induction of oxidative stress, perturbation of the epithelial cells proliferation/apoptosis ratio, and cytokine secretion [7]. In this study, our data indicate that CD-14 overexpression also modulates the invasive potential of cells. Although the molecular basis behind this effect requires further investigation, it is likely associated with immunogenetic factors, which play important roles in inflammation progression and severity, thereby influencing the clinical outcome of *H. pylori* infection. We speculate that CD-14 overexpression activates NF-κB transcriptional activity to induce pro-inflammatory cytokine secretion, thus causing alterations in the microenvironment. Microenvironment changes constitute a key event in promoting cancer development and progression. However, we cannot rule out the involvement of modulators beyond the scope of this study, which will be the focus of further studies. In addition, the activation of the NF-κB-mediated signaling is known to protect cancer cells from undergoing apoptosis. But, we discovered the increased apoptotic cells in CD-14 overexpressing cells regardless of the upregulated NF-κB activity. The molecular basis behind this observation is unclear at this point. We speculate that NF-κB signaling only partially contributes to the ultimate fate of the cells. Mechanism beyond this pathway may be affected by CD-14 upregulation and deserves further studies.

In summary, we have provided experimental evidence to elucidate the tumor-suppressive role of CD14 in gastric cancer. CD14 overexpression results in reduced clonogenic ability, proliferation, and metastatic invasion as well as enhanced apoptosis. These effects may be partially due to activated NF-κB by simultaneously upregulating its expression and stimulating its DNA binding activity, which thereby activates a series of pro-inflammatory cytokines, including TNF-α, IL-1β, IL-6, and IL-12.
